# Bacterial Genetic Engineering by Means of Recombineering for Reverse Genetics

**DOI:** 10.3389/fmicb.2020.548410

**Published:** 2020-09-11

**Authors:** Ursula Fels, Kris Gevaert, Petra Van Damme

**Affiliations:** ^1^Department of Biochemistry and Microbiology, Ghent University, Ghent, Belgium; ^2^VIB-UGent Center for Medical Biotechnology, Ghent, Belgium; ^3^Department of Biomolecular Medicine, Ghent University, Ghent, Belgium

**Keywords:** bacterial genetics, enterobacteriaceae, (phage-based) homologous recombination, precise genome editing, recombineering, reverse genetics, selection markers

## Abstract

Serving a robust platform for reverse genetics enabling the *in vivo* study of gene functions primarily in enterobacteriaceae, recombineering -or recombination-mediated genetic engineering-represents a powerful and relative straightforward genetic engineering tool. Catalyzed by components of bacteriophage-encoded homologous recombination systems and only requiring short ∼40–50 base homologies, the targeted and precise introduction of modifications (e.g., deletions, knockouts, insertions and point mutations) into the chromosome and other episomal replicons is empowered. Furthermore, by its ability to make use of both double- and single-stranded linear DNA editing substrates (e.g., PCR products or oligonucleotides, respectively), lengthy subcloning of specific DNA sequences is circumvented. Further, the more recent implementation of CRISPR-associated endonucleases has allowed for more efficient screening of successful recombinants by the selective purging of non-edited cells, as well as the creation of markerless and scarless mutants. In this review we discuss various recombineering strategies to promote different types of gene modifications, how they are best applied, and their possible pitfalls.

## Introduction

**FIGURE 1:**
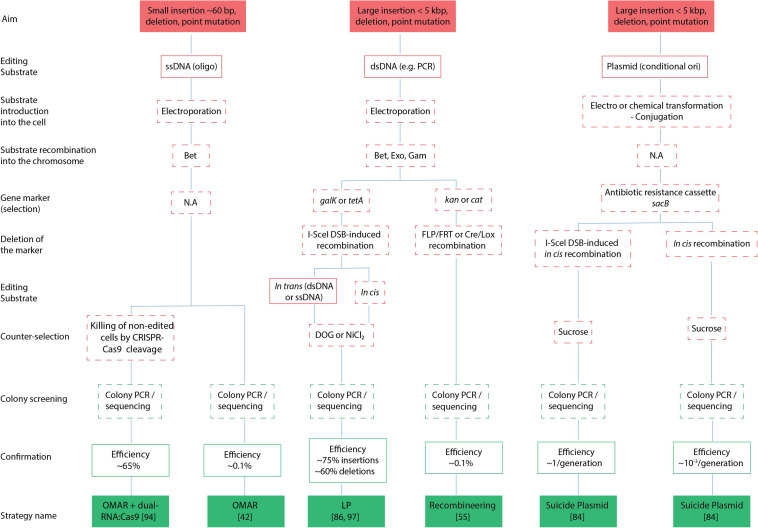
Flowchart of genetic engineering strategies. Schematic representation of different strategies (filled green boxes) categorized by their overall aim (filled red boxes), editing substrates to be used (red boxes), (counter) selection (red dashed line), screening (green dashed line) and efficiencies of attaining successful transformants (green boxes). N.A, not applicable; bp, base pair; kbp, kilo base pair; dsDNA, double stranded DNA; ssDNA, single stranded DNA; DSB, double strand break.

With the advent of next-generation sequencing, there has been an exponential rise in the number of complete bacterial genome sequences that were made publicly available (Land et al., 2015), making it necessary to increase efforts to correctly annotate and assign gene functions. The latter task is mostly achieved by reverse genetic approaches that consist of altering the gene sequence e.g., deletion, tagging, reporter gene fusion or by introducing base pair (bp) changes, to determine its function by phenotypic analysis. Genome editing of bacteria is typically attained by homologous recombination between the target gene and an editing substrate that can either be circular or linear DNA, the latter being single-stranded DNA (ssDNA) oligonucleotides (oligos) or double-stranded PCR products (dsDNA). These editing substrates are introduced in the bacteria using transformation, conjugation or transduction, and we refer to Snyder et al. (2013) for more detailed information on these different strategies. Homologous recombination between the target gene and the editing substrate can be achieved by endogenously expressed recombination genes (e.g., allelic exchange by cointegrate formation and resolution), the use of recombination-proficient strains, or alternatively, by phage recombination systems (i.e., recombineering). Particularly, the use of phage recombineering proteins to perform recombineering was proven to be a highly efficient method to modify not only bacterial chromosomes, but also other replicons such as bacterial artificial chromosomes (BACs), which are frequently used to accommodate large inserts [up to several hundred kilo base pairs (kbp)]. In particular, *Escherichia coli* (*E. coli*) and *Salmonella enterica* (*S. enterica*) have extensively been used as workhorses for the implementation and development of different DNA-based recombineering technologies (Cronan, 2014). While recombineering strategies will be the main focus of this review, bacterial gene replacements historically involved allelic exchange methodologies, and thus will also be briefly discussed. Such approaches still remain critical for modifying bacteria where phage recombination systems are not yet available, or where DNA transformation protocols are inefficient. While previous excellent reviews on this topic have been published (Court et al., 2002; Thomason et al., 2007, 2014; Murphy, 2016), we here focus on the applicability of recombineering for reverse genetics, and discuss the most recent developments in the field of bacterial recombineering. A flowchart of the strategies highlighted in our review is provided in Figure 1.

## Selection and Counter-Selection Recombineering Strategies

The very first and common step of most genome engineering strategies is the introduction of exogenous DNA elements (e.g., plasmids, dsDNA or ssDNA), which is often achieved by transformation. While transformation is typically more convenient than other strategies (e.g., conjugation for large-scale studies), in the case of commonly used electrical transformation (i.e., electroporation), only up to 5% of input cells are transformed when making use of plasmid DNA (Wu et al., 2010). Moreover, exact efficiencies of transformation are very difficult to report since they depend on many variables such as residual salt or charged chemicals prior to electroporation, the number and density of electroporated cells, electroporation volume, survivors of electroporation, type and input of DNA and the growth phase among others (Wu et al., 2010).

Besides considering efficiencies at the individual steps of a recombination strategy, there is an inherent efficiency limitation. As discussed in Pines et al. (2015), the theoretical maximum recombination efficiency in bacteria is mainly determined by bacterial chromosome segregation during cell division, requiring up to two replication rounds for complete segregation of the introduced modification to one of four daughter cells. Furthermore, exponentially growing bacteria normally contain up to 8 copies of their chromosome, which decreases this efficiency even further. Taken together, when considering that 5% of input cells are transformed and that only 3–6% of these will contain the desired modification, the maximum efficiency of recombination is only ∼0.15–0.3%. Therefore, to avoid extensive and time-consuming screening of transformed bacteria in an exceedingly non-edited background, co-transformation with different drug resistance (drug^*R*^) markers is frequently used to select for transformed bacteria. These markers can be classified as positive selectable, when upon selection they confer an advantage to the transformants over non-edited cells, or as negative (or counter-selectable) when they eliminate or inhibit growth of the transformed cell. Some commonly used gene markers and their genetic background compatibility are summarized in Table 1.

**TABLE 1:** Commonly used gene markers for selection and/or counter-selection of modified over non-edited bacteria.

**Selectable marker gene (abbreviation)**	**Encoded enzyme**	**Substrate used for selection**	**Substrate used for counter-selection**	**(Original) gene source**	**Strain genetic background**
**Commonly used antibiotic resistance cassettes**					
*neoR, kan, nptII*	Neomycin phosphotransferase II	Kanamycin	N.A.	Transposon Tn5 (*Escherichia coli*) (Reiss et al., 1984)	*N.A*
*cat*	Chloramphenicol acetyltransferase	Chloramphenicol	N.A.	*Escherichia coli* (Shaw et al., 1979)	*N.A*
*bla*	Beta-lactamase	Ampicillin	N.A.	*Escherichia coli* (Ambler and Scott, 1978)	*N.A*
*tetR*	Tetracycline repressor protein class C	Tetracycline	Fusaric acid or NiCl_2_	*Escherichia coli* (Podolsky et al., 1996; Nefedov et al., 2000)	*N.A*
*tetA*	Tetracycline resistance protein, class C	Tetracycline	Fusaric acid or NiCl_2_	*Escherichia coli* (Maloy and Nunn, 1981; Podolsky et al., 1996)	*N.A*
**Metabolic markers**					
*galK*	Galactokinase	Minimal media with galactose as unique carbon source	2-deoxy-galactose (DOG)	Alper and Ames, 1975	Δ*galK*
*thyA*	Thymidylate synthase	Media lacking thiamine	Trimethoprim	Stacey and Simson, 1965	Δ*thyA*
*pyrE*	Phosphoribosyl transferase	Media lacking uracil	5-fluoroorotic acid (5-FOA)	Sakaguchi et al., 2013	Δ*pyrE*
*pyrF*	Decarboxylase	Media lacking uracil	5-fluoroorotic acid (5-FOA)	Galvao and de Lorenzo, 2005	Δ*pyrF*
*upp*	Phosphoribosyl transferase	Media lacking uracil	5-fluoroorotic acid (5-FOA)	Fabret et al., 2002	Δ*upp*
**Other markers**					
*tolC*	TolC (outer membrane protein)	Sodium dodecyl sulfate (SDS)	Colicin E1	DeVito, 2008	Δ*tolC*
*sacB*	Levansucrase	N.A.	Sucrose	*Bacillus subtilis* (Gay et al., 1985)	*N.A*
*kil*, repressor *cI857*	Kil	30°C (*cI857* active)	42°C (*cI857* inactive)	*Bacteriophage lambda* (Chen et al., 2020)	*N.A*
*pheS* Gly204	Mutant tRNA synthetase	N.A	Chloro-phenylalanine	Li and Elledge, 2005	*N.A*
*ccdB-bla* and *ccdA*	Toxin CcdB/Antitoxin CcdA	Ampicillin	42°C (outflow of *ccdA*)	Wang H. et al., 2014; Zhang et al., 2017	*N.A*

Phenotypic screening markers can be used as an alternative to drug^*R*^ selectable markers. Expression of chromoproteins such as *amilCP* or magenta *tsPurple* results in blue and purple colonies respectively, enabling selection by colony coloring (Lazarus et al., 2019). While a palette of engineered chromoproteins was recently assessed in *E. coli*, it is noteworthy that optimal conditions for attaining intense coloring, low fitness cost, and fast maturation have not yet been described (Liljeruhm et al., 2018). In the context of recombineering editing substrates, the alpha subunit from *lacZ* (*lacZ’*) has been used to restore the LacZ + phenotype when delivered into LacZ^–^ cells (*lacZ*Δ*M15*, Yanisch-Perron et al., 1985) known as alpha complementation (Ullmann et al., 1967). Using this assay, visual inspection of colonies on LB agar plates supplemented with X-Gal substrate allow differentiation of blue transformed colonies from white non-transformed colonies, which are unable to metabolize X-Gal (Pyne et al., 2015). Other reporter genes that can be used to identify transformants, are green fluorescence protein (GFP), DsRed and luciferase (Gerlach et al., 2007).

Several markers are appropriate for positive selection of transformants, the most widely used being antibiotic resistance markers, mostly because selection is easily achieved by supplementing the media with the desired antibiotic. Some bacterial antibiotic selection markers that have been used extensively are genes conferring resistance to kanamycin (*kan* gene), chloramphenicol (*cat* gene), ampicillin (*bla* gene, beta-lactamase) and tetracycline (*tetR* and *tetA* genes) (Table 1). Another strategy uses *tolC*, encoding the outer membrane protein TolC that harbors efflux transmembrane transporter activity, which confers an increased resistance to toxic molecules such as sodium dodecyl sulfate (SDS), while its loss confers resistance to the membrane depolarization activity of colicin E1, allowing for its direct counter-selection (DeVito, 2008). Another example is *tetA*, the removal of which can also be counter-selected with fusaric acid or NiCl_2_, as these compounds are selectively lethal to cells expressing *tetA* (Maloy and Nunn, 1981; Podolsky et al., 1996). Some genes are only counter-selectable and thus do not require a particular genetic background, e.g., *Bacillus subtilis sacB*, and have therefore been frequently used in combination with a positive selectable marker (Li et al., 2013). Of note, a limitation of *sacB* counter-selection is acquired bacterial resistance to its toxic product given the high spontaneous *sacB^+^ to sacB*^–^ conversion rate observed (10^–4^) (Hmelo et al., 2015). Another example of counter-selection is the use of the bactericidal phage gene *kil* under the control of a temperature-sensitive repressor *cI857*. Shifting the temperature to 42°C results in repressor inactivation and concomitantly, killing of bacteria expressing *kil* (Chen et al., 2020). The A294G mutant of *E. coli* phenylalanyl-tRNA synthetase (*pheS*^*Gly*294^) is another counter-selection marker that can be used, as the mutant PheS permits 4-chloro-phenylalanine incorporation in proteins, which is toxic for cells (Li and Elledge, 2005). The use of the *ccdB* toxin/*ccdA* antitoxin system constitutes an efficient counter-selection strategy for the selection of recombinant cells by insertion of the *ccdB-bla* cassette (media supplemented with ampicillin) while expressing antitoxin *ccdA* from a temperature-sensitive Red-producing plasmid. Switching to non-permissive temperatures (42°C) results in the loss of *ccdA* encoding plasmid and only cells that successfully exchanged the *ccdB* cassette by a second recombineering event survive (Wang H. et al., 2014; Zhang et al., 2017).

Incorporation of so-called metabolic markers (Table 1) enables transformants to grow in the presence of different carbon sources or in media lacking an otherwise essential nutrient. However, the use of metabolic markers requires, besides an appropriate genetic background, the use of defined or minimal media in which bacteria often grow poorly (Li et al., 2014). An example of a metabolic marker is *galK*, as *galK* transformants can be selected by growing with galactose as the unique carbon source. Counter-selection with *galK* is performed in media supplemented with 2-deoxy-galactose (DOG), as upon phosphorylation of DOG by GalK, the toxic compound 2-deoxy-galactose-1-phosphate is produced (Alper and Ames, 1975). Another commonly used metabolic marker *thyA*, encodes the enzyme thymidylate synthase (ThyA) transformants of which can be selected for in media lacking the essential nutrient thiamine and counter-selected with trimethoprim, the latter acting as an inhibitor of dihydrofolate (DHF) reductase (Stacey and Simson, 1965; Stringer et al., 2012). DHF is responsible for replenishing the levels of tetrahydrofolate (THF), an essential cofactor for many cellular processes, including ThyA functioning. Those cells containing *thyA* rapidly consume the pool of THF, and ensuing growth is suppressed due to incapability to replenish THF. On the contrary, in cells without *thyA*, the THF pool is maintained for other cellular reactions, and thus the absence of *thyA* can be selected for on minimal media plates containing both thiamine and trimethoprim (Wong et al., 2005). Clearly, the use of *galK* and *thyA* as both selection and counter-selection markers is restricted to strains that do not encode functional copies of these genes on their chromosomes. As such, the use of both *thyA* and *galK* as markers have most commonly been applied for the manipulation of plasmids and bacterial artificial chromosomes (BAC), where transformation of these episomes into *E. coli* must be performed as a first step. The use of *thyA* or *galK* strains (in place of wild type *E. coli*) thus does not require an additional step for making genetic manipulations of episomes (Warming et al., 2005; Wong et al., 2005).

In this context, it is however noteworthy that, while a Δ*thyA* strain – promoted as a standard strain for permitting a more versatile and efficient recombination in enterobacteria (Stringer et al., 2012) – was successfully created in the *S. enterica* strain 14028s (Stringer et al., 2012), we and others were unable to attain a Δ*thyA* deletion mutant in the context of *S. enterica* strain SL1344 (personal communication Prof. Joseph Wade (Wadsworth Center, New York, United States of America) and our unpublished observation), which likely indicated its essentiality in this specific *S. enterica* strain, as *thyA* was reported among the 343 essential SL1344 genes when grown in rich medium (Barquist et al., 2013). Other metabolic markers broadly used in bacteria (Fabret et al., 2002; Galvao and de Lorenzo, 2005; Sakaguchi et al., 2013) include *E. coli pyrE* and *pyrF* or the *B. subtilis pyrE* ortholog *upp*, all implicated in the pathway of pyrimidine synthesis and counter-selectable in strains with the corresponding genetic background (e.g., Δ*pyrE*, Δ*pyrF* or Δ*upp*). Bacteria harboring a copy of any of these markers are able to grow in the absence of uracil and are sensitive to 5-fluoroorotic acid (5-FOA) given its metabolization to the highly toxic compound 5-fluorouridine monophosphate (5-FMP).

## Gene Insertion, Deletion or Mutation via Endogenous Recombination Systems

**FIGURE 2:**
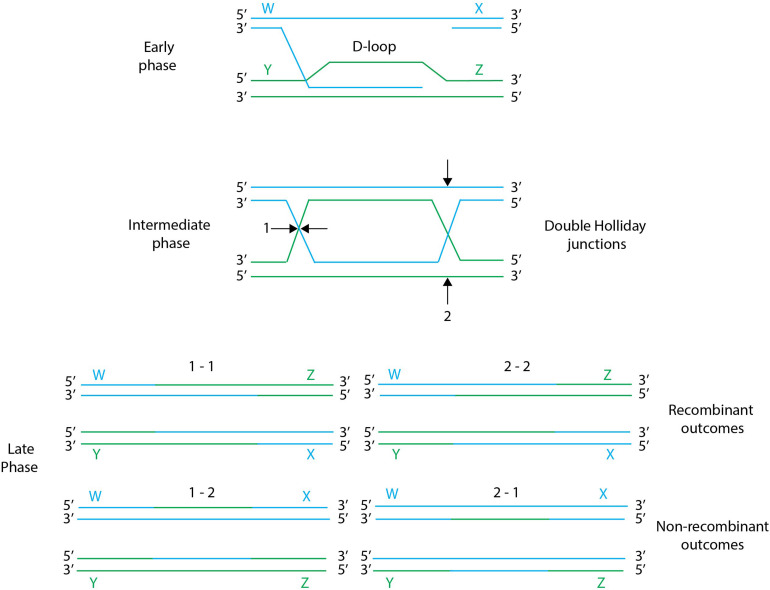
General scheme of homologous recombination. Early, intermediate and late phases of homologous recombination: in the early phase the ssDNA arm from a nicked chromosome invades a homologous chromosome forming a D-loop, the resulting branched DNA leads to the formation of a double Holliday Junction intermediate. Holliday junctions can be resolved during the late phase by two types of resolution cuts, designated 1 and 2, resulting in different recombination outcomes. Resolution of Holliday junctions by the same cuts (i.e., 1–1 or 2–2) results in recombinants molecules, while resolution of Holliday junctions by the different cuts (i.e., 1–2 or 2–1) results in wild-type molecules with parental shoulders. Figure adapted from Kuzminov (2011).

Gene editing by homologous recombination involves the exchange between homologs DNA sequences in a process catalyzed by recombination systems, each characterized by its minimal length of DNA homology required. Homologous recombination is a complex process, where the DNA exchange mechanisms consist of early, intermediate and late phases. While the early phase consists of the end-processing and invasion of ssDNA from one DNA duplex into another to form a D-loop, the intermediate phase consists of the formation of branched DNA that eventually form recombination intermediates know as Holliday junctions. In the final phase, the Holliday junctions are cut by resolvases to generate recombinant chromosomes, either with or without crossover of flanking sequences (Figure 2). Different resolution cuts at the left or the right of the junctions result in four possible outcomes: two recombinant molecules with exchange of flanking sequences, or two patch recombinants with no exchange of flanking sequences. Note that while there are several types of branched DNA molecules, for the sake of simplicity double Holliday junctions are shown in Figure 2. For details on resolution mechanisms we refer to Kuzminov (2011).

A suicide plasmid cannot replicate under certain conditions (e.g., host incompatibility, non-permissive temperatures), and thus only its integration into the chromosome by homologous recombination prevents its loss. This mechanism of integration depends on endogenous homologous recombination systems and is the first event leading to a classical mechanism of allelic exchange. Its application greatly facilitated chromosomal gene replacement and the generation of knockouts, and is of central importance for reverse genetic-assisted discoveries in many Gram-negative bacteria. This strategy was first established in 1989 by Hamilton et al. (1989), who reported the use of a plasmid with a thermosensitive conditional replication origin and antibiotic resistance cassette to replace a gene with a mutated allele. Transformed bacteria that inserted the plasmid DNA sequence into their chromosome, known as co-integrants or merodiploids, were formed at non-permissive temperatures by homologous recombination between the chromosomal wild-type gene and the mutated gene cloned in the suicide plasmid, a process referred to as recombination *in trans*. After selection of antibiotics-resistant co-integrants, a second *in cis* or intrachromosomal recombination event – between the two duplicated copies of the gene (wild-type and mutated) – can occur at permissive temperatures, leading to the resolution of the co-integrant, an event which can be selected by the screening for sensitivity to the antibiotic used in the selection of the co-integrant.

An improved version of this strategy (Link et al., 1997) identified the resolution of recombinants using *sacB* counter-selection by the killing of non-resolved co-integrants upon sucrose addition. Besides allelic replacement, gene insertions and deletions can be generated using this strategy (Lee et al., 2007; Winkler and Kao, 2012; Sahu et al., 2013). Of note, in difficult-to-transform bacterial strains (e.g., *Serratia marcescens*), conjugation constitutes an alternative approach to introduce the plasmid DNA editing substrates (Snyder et al., 2013). Here, the plasmid DNA is transferred from a donor strain to a recipient strain in a process dependent on a pilus structure (Lazarus et al., 2019).

The main drawback of the use of allelic exchange approaches is the need to perform extensive screening (e.g., colony PCR, sequencing or phenotypic testing) to select those transformants that resulted in the resolution outcome that contains the desired change in the chromosome (i.e., did not re-generate the wild-type genetic sequence), making it an inefficient and time-consuming procedure. Furthermore, endogenous host cell recombination systems require large homology arms (see below), which also make the often tedious sub-cloning and construction of lengthy editing sequences in appropriate vectors a prerequisite.

## Recombineering: Genetic Editing by Means of Phage Recombination Functions

Since allelic exchange makes use of dsDNA allelic exchange substrates, cloning using linear DNA substrates is more straightforward as it requires fewer DNA manipulations and the introduction of the homologous substrate is usually provided in the form of PCR products or synthetic oligos. However, as part of the bacterial defense system, bacterial transformation with linear DNA typically results in DNA degradation by endogenous nucleases. More specifically, while bacterial DNA is protected from nuclease action by sequence-specific DNA methylation introduced by the action of DNA methyltransferases (Marinus and Løbner-Olesen, 2014), non-methylated incoming linear exogenous DNA is cut by bacterial nucleases.

Besides their role in bacterial defense systems and common use for the purpose of molecular cloning (e.g., *Eco*RI and *Bam*HI), exo- and endonucleases are also crucial for DNA repair and recombination functions. In view of the later, removal of restriction systems was shown to be important for getting good recombination efficiency (Mosberg et al., 2012) and scientists struggled for many years to knock-out the important restriction systems to “domesticate” their favorite organism. For example, the domesticated MG1655 derived DH5α *E. coli* strain contains inactivating mutations in the endonuclease genes *endA* and *hsdR17*, thereby facilitating cloning and recombineering, next to improving plasmid yield (Woodcock et al., 1989; Chen et al., 2018). As nuclease activity and recombineering efficiency are thus heavily intertwined, we refer to Lovett (2011) for a more extensive description on the function and characterization of nucleases involved.

With its high affinity toward dsDNA ends (Taylor and Smith, 1985; Roman and Kowalczykowski, 1989), the RecBCD nuclease was shown to be the main actor in degrading linear exogenous DNA. RecBCD, formed by RecB, RecC, and RecD possesses DNA helicase and exo- and endonuclease activity, and plays a role in *recA* mediated recombination, a process mainly implicated in DNA damage repair. Historically, one strategy to promote recombination with linear DNA was the use of a recombination-proficient strain carrying null mutations of the *recB* (*recB22*) and *recC* (*recC21*) genes; these mutants lacked nuclease activity and were deficient in recombination. By selection of suppressor mutations in *recB22 recC21* strains that restored recombination proficiency, it was found that inactivation of the *sbcBC* genes (suppressor of *recBC* mutations) activated an alternative recombination pathway that was dependent on RecF activity, and became known as the RecF pathway (Kushner et al., 1971; Lloyd and Buckman, 1985). Later on, it was discovered that uniquely mutating *recD* resulted in the creation of a nuclease-deficient recombination-proficient strain (Russell et al., 1989). However, the use of this mutated strain often led to unwanted recombination events and subsequent genome instability. However, the advent of phage recombineering systems not only greatly improved recombination efficiencies and reduced homology length requirements, but additionally permitted the expression of recombineering proteins from inducible promotors, thereby avoiding genome instability (see below).

In 1998, the action of the recombination proteins from the bacteriophage lambda (λ) *red* system were reported to outperform the recombination efficiency of the traditionally used Δ*recBCD sbcBC o*r *recD* mutant *E. coli* strains as up to 130-fold when using linear products with large homology arms as editing substrates (Murphy, 1998). Later in that same year, the Rac prophage recombination functions RecET were reported to promote gene replacement with homology arms as short as 40 bp, ∼10-fold shorter as compared to homology arms used in case of recombination performed when making use of *E. coli* recombination-proficient strains with endogenous mutated recombinases (Zhang et al., 1998). While the homology requirements needed for recombination in the context of phage infection are still unknown, shortening of homology arms represented a breakthrough in genetic engineering, as it led to the use of PCR-generated linear substrates containing short flanking homologies as allelic exchange substrates, thus avoiding the need for sub-cloning of long homology arms for the construction of editing substrates. Since their introduction, and in light of their increased performance over endogenously-derived recombination-proficient bacterial strains, phage recombineering systems have widely been adopted for genome engineering in the last 20 years.

Bacteriophage λ-mediated recombineering involves the expression of the bacteriophage λ-encoded genetic recombination machinery, named the λ *red* system, consisting of the *exo* and *bet* genes, and assisted by the *gam* gene. Together, these genes are designated λ *red* genes. Noteworthy, Rac prophage recombineering genes, *recE* and *recT* found in *E. coli*, encode non-homologs but functionally equivalent products as *exo* and *bet* genes, respectively (Clark et al., 1993). The product of the *gam* gene, the Gam protein, inhibits RecBCD and SbcCD exonuclease activities (Sakaki et al., 1973; Kulkarni and Stahl, 1989; Murphy, 1991), thereby avoiding degradation of linear exogenous DNA. Exo is a 5′3′ exonuclease which targets dsDNA and Bet is a ssDNA-binding protein that promotes the annealing of complementary ssDNA strands. When bound to ssDNA, Bet protects its substrate from degradation. Exo, Bet and Gam thus promote homologous recombination between a target gene and a linear dsDNA PCR fragment containing a selection marker or, alternatively, a ssDNA oligo, with both substrates containing short homology regions to the target region.

While the molecular details of the mechanism of λ *red* homologous recombination are not yet completely deciphered, evidence so far indicates that it can occur via at least two distinct pathways: one being *recA*-dependent and replication-independent or so-called strand invasion, and one being *recA*-independent and replication-dependent or so-called strand annealing (Stahl et al., 1997; Murphy and Marinus, 2010; Poteete, 2013). In the context of recombineering, there is conclusive evidence that λ *red* recombination between exogenous and chromosomal DNA occurs at the replication fork, preferentially through the interaction with the lagging strand template (Lim et al., 2008). This was illustrated by Lim et al. (2008) who inserted non-homologous regions with progressively increasing length at the end of oligos corresponding to both the lagging or the leading strand of the replication fork. They observed that non-homologous sites attached to an oligo corresponding to the lagging strand reduced the recombination efficiency ∼7-fold. Other oligo-based studies also showed that recombination occurs with a higher efficiency (up to ∼30-fold) via annealing of oligos to the lagging strand template (Ellis et al., 2001; Costantino and Court, 2003; Li et al., 2003; Sawitzke et al., 2011). Based on these observations, the discontinuous nature of the lagging strand appears to accommodate the efficient annealing of ssDNA oligos to the lagging strand template better relative to the leading strand template, presumably because of the presence of gaps in the leading strand. For more details of λ *red* recombineering and its proposed molecular mechanisms, we would like to refer to (Murphy, 2016).

To date, three different ways of expressing λ *red* genes have been reported, namely plasmid, chromosome or defective λ prophage-based expression. All of them rely on the tightly controlled expression of the λ *red* recombination genes from strong inducible promotors (which are induced for expression by either IPTG, arabinose or a shift in temperature). Tight control in *red* expression is important to avoid undesirable secondary mutations (and thus genome instability) as spontaneous generation of antibiotic-resistant colonies were reported in *E. coli* when constitutively expressing λ *red* recombination genes (Murphy and Campellone, 2003). Moreover, due to inhibition of RecBCD (Sergueev et al., 2001), Gam expression also infers a certain degree of toxicity to the cells. In the case of Rac recombineering functions, expression of *recE* and *recT* genes from the endogenous Rac prophage only gives low levels of recombineering, thus requiring expression from multicopy plasmids under the control of heterologous promoters (e.g., the arabinose-inducible pBAD promoter; Zhang et al., 1998) to promote efficient gene replacement.

The λ *red* system was first implemented by Murphy (1998), who expressed *exo*, *bet* and *gam* either from a high copy plasmid or from the chromosome. Based on the belief that phage recombination systems require long homology lengths, the flanking homology sequences used in this study were lengthy, between ∼1000 and 3500 bp. Surprisingly, despite the lower expression of the λ *red* system, Murphy’s work (Murphy, 1998) indicated a 4-fold increase in the recombination frequencies when *exo*, *bet* and *gam* were expressed from the chromosome, a compensation explained by the absence of competitive plasmid multimers which inhibit the recombination process. Later on Datsenko and Wanner (2000) shortened the homology regions used for recombination to 36–50 bp and expressed *exo*, *bet* and *gam* from the low copy helper plasmid, pKD46. More specifically, pKD46 contains a temperature sensitive origin of replication, which facilitates its later curing by switching to non-permissive temperatures. In this context, plasmid clearance was proven essential to avoid secondary mutations such as large random deletions (deletion up to 17 kbp were demonstrated) (Hobman et al., 2007).

The λ *red* genes can also be expressed under their native control from a defective prophage in which the operon structure encoding the DNA replication genes, lysis cassette and structural genes is deleted (Yu et al., 2000; Ellis et al., 2001). In strains containing this defective prophage, e.g., DY380 (Lee et al., 2001), *exo*, *bet* and *gam* genes are expressed from the endogenous p_*L*_ operon under control of *cI857*, which encodes a temperature-sensitive version of the cI repressor that is only functional at low temperatures (below 34°C). Its inactivation is therefore possible by shifting the temperature to 42°C with the concomitant expression of *exo*, *bet* and *gam* genes. To introduce λ *red* functions in the desired strain, mini-λ (Court et al., 2003) circular non-replicating phage DNA can be introduced into any strain, where it is stably integrated into the chromosome by site-specific recombination, and thus without requiring selection for maintenance. By the introduction of an antibiotic resistance cassette flanked by 50 bp homology regions, gene knock-outs were achieved with an efficiency of ∼0.1% (i.e., up to 1 transformant per 1000 electroporated cells). A study reporting the use of plasmids with a different origin of replication expressing *exo*, *bet* and *gam* genes under their native control (pSIM plasmids) achieved similar efficiencies of gene knock-outs in *E. coli* and *S. enterica* as those reported for the prophage strain (Datta et al., 2006), while up to 10 to 100- fold higher efficiencies were reported for these strains when compared to making use of the pKD119 plasmid (Datsenko and Wanner, 2000), a pKD46 related plasmid with a different selection marker.

The RecET recombination system was implemented for the first time by Zhang et al. (1998), additionally requiring the co-expression of *gam* from λ *red* to inhibit RecBCD activity. Since RecE is ∼4-fold larger than its functional homolog Exo (Kovall and Matthews, 1997; Zhang et al., 2009), only its C-terminal domain has typically been used. Nevertheless, full length RecE (along with RecT and λ Gam) was shown to promote recombination between linear dsDNA species at considerably higher efficiencies than λ *red* recombineering (Fu et al., 2012), making it the better alternative for incorporation of linear dsDNA into a linear plasmid (Thomason et al., 2014).

## Strategies for Removal of Selection Markers

**FIGURE 3:**
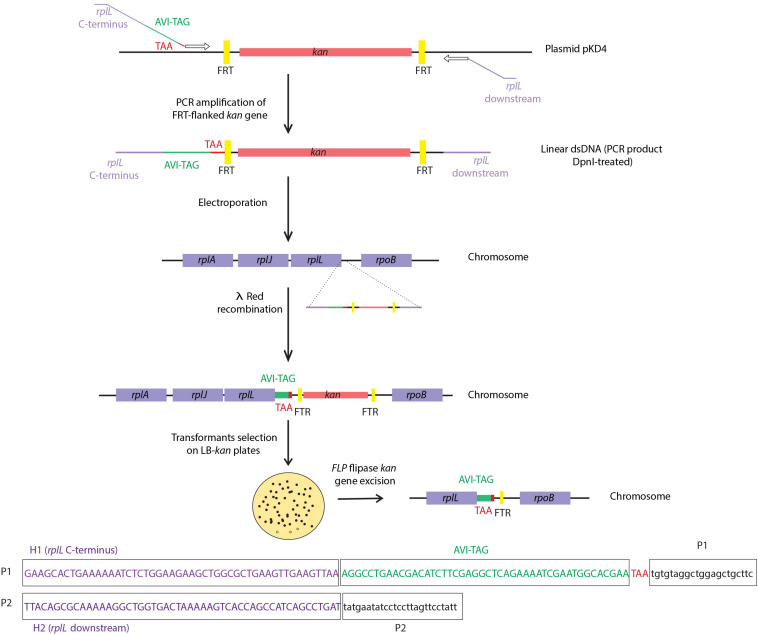
Tagging of the chromosomal *rplL* gene in *Salmonella enterica* strain SL1344 and FLP-mediated excision of the antibiotics resistance cassette. The resistance marker *kan*, flanked by FRT regions, is PCR-amplified from the plasmid pKD4 by making use of primers P1 and P2, with *rplL* C-terminus and downstream homology regions (H1 and H2), respectively. Primer P1 also contains a tag (i.e., AVI-tag) sequence followed by a translation stop codon (TAA). The resulting linear dsDNA PCR product flanked with sequences homologous to the target region is electroporated into competent *S. enterica* expressing λ *red* recombination genes. Upon selection of kanamycin resistant transformants, flippase (FLP) mediated *kan* gene excision is induced, leaving an FRT scar sequence. In line with the tagging of *E. coli rplL* performed in Ederth et al. (2009), tagging of *rplL* of *Salmonella enterica* strain SL1344 was performed *in house*.

In case of successful transformation, removal of selection markers is desirable to avoid possible transcriptional perturbation due to the presence of the marker’s promoter. This removal can be achieved by the *Saccharomyces cerevisiae* flippase (FLP), a site-specific recombinase that catalyzes excision of DNA between two tandem FLP recombination target sequences (FRTs) (Sherman, 1982). This process is illustrated in Figure 3, where after tagging of the *rplL* gene, the kanamycin selection marker is removed. Since FLP-mediated excision requires FRT sites flanking the selection cassettes, there are several plasmid templates that can be used for PCR-based amplification of the linear PCR fragments to be used as substrates for recombineering [e.g., pKD3 and pKD4 plasmids (Datsenko and Wanner, 2000) containing FRT sites flanking either a chloramphenicol or a kanamycin resistance cassette, respectively]. Routinely, to prevent the generation of false positives, PCR products are treated with *Dpn*I restriction enzyme, which selectively digests methylated parental DNA templates, or alternatively, when selection markers are chromosomally located, colony-PCR can be used for obtaining such linear PCR-fragments. Furthermore, the conditional replication origins of the commonly used plasmids averts plasmid persistence in case of incomplete *Dpn*I digestion.

Upon FLP-mediated excision of such antibiotic resistance cassettes (from pKD3 or pKD4), a sequence of ∼85 bp, generally referred to as a scar sequence, remains in the chromosome. To avoid undesired effects on expression of downstream genes, the scar sequence contains stop codons in all the six reading frames, a ribosome binding site (RBS), and a start codon for downstream translation in polycistronic transcription units. This scar may vary in length dependent on the template plasmid chosen, with the minimal scar size reported being 36 bp (Senecoff et al., 1988). In case of the pCP20 helper plasmid, the *FLP* gene is expressed from the λ *p*_*R*_ promoter under the control of *cI857* (Cox, 1983), and has a thermosensitive replication origin to facilitate its cure upon FLP-mediated excision (Cherepanov and Wackernagel, 1995).

A major limitation of this strategy arises when targeting multiple genes due to possible recombination between existing and incoming FRT sequences, or the occurrence of *in cis* recombination between FRT scars. To overcome this situation, a similar system consisting of the bacteriophage P1 Cre/*loxP* has been used to excise selection markers (Nagy, 2000). Unlike FRT sites, variants of *loxP* sites (e.g., *loxLE*, *lox2272*) can be used when multiple gene targeting is needed. While still being recognized by the Cre recombinase, the simultaneous presence of multiple variant *loxP* sites in the genome will remain, without the occurrence of undesired recombination events (Carter and Delneri, 2010).

Following the original Datsenko protocol (Datsenko and Wanner, 2000), single gene modification and subsequent antibiotic cassette removal takes ∼7 days, considering the sequential introduction and subsequent removal of temperature sensitive helper plasmids, pKD46 and pCP20. However, by merging both set of genes required for recombination and selection marker excision into a single helper plasmid, the time was reduced to only 3 days by alleviating the need of multiple curing and transformation steps (Song and Lee, 2013; Jensen et al., 2015). This protocol was further shortened to complete 7 sequential gene knock-outs in only 7 days, including the removal of antibiotics resistance cassettes (Jensen et al., 2015). To achieve this, λ *red* and *FLP* genes together with their respective promotors were cloned in the chromosome, which enabled the recovery and growth at permissive temperatures (37°C instead of 30°C) throughout the process. Furthermore, *FLP* induction times were shortened from overnight (∼18 h) to only 4 h, and the use of two different resistance cassettes allowed the simultaneous targeting of two genes.

## Oligo-Mediated Allelic-Replacement (OMAR) Avoids the Use of Selection Markers

Instead of using PCR-generated dsDNA molecules containing a selectable marker and flanking homology regions that reach efficiencies of about 0.05%, synthetic short ssDNA oligos (50–90 nt.) can also be used to generate small base substitutions and insertions (up to ∼30 bp), as well as (larger) deletions (up to ∼45 kbp). Even though the general scope is thus lower compared to using dsDNA, the efficiency of OMAR outperforms dsDNA-based recombineering reaching up to 25% of surviving cells after electroporation when methyl-directed mismatch repair system (MMR) is inactivated or bypassed (see below) (Costantino and Court, 2003). Thus, the use of OMAR eliminates the need for selection as only a manageable number of colonies have to be tested for finding successful recombinants. Further, the use of ssDNA substrates for λ *red* recombineering only requires Bet for recombination, which delivers ssDNA oligos to the replication fork where they anneal with the lagging strand template (Costantino and Court, 2003).

Ellis et al. (2001) implemented the use of ssDNA for the first time for correcting an amber mutation in *galK* which generated a premature stop codon (Gal^–^ phenotype). Cells were selected for restoration of the Gal^+^ phenotype, obtaining 0.1% recombination efficiency (Figure 1) when making use of 70 bases long ssDNA. When using 40–60 bases long ssDNA, this efficiency dropped ∼5-fold, and with 30 bases long ssDNA another ∼10-fold, until reaching background levels when using 20 bases long ssDNA (Ellis et al., 2001). Consistent with *in vitro* experiments showing that Bet binds weakly to sequences shorter than 36 bases (Mythili et al., 1996), decreasing the oligo length below 40 bases showed an exponential decrease in recombination efficiency (Sawitzke et al., 2011). Increasing the length to 90–120 bases however, showed only a ∼2 fold higher overall efficiency (Wang et al., 2009), a very modest increase when considering the significantly higher synthesis and purification cost of longer oligos, as well as the increased probability of introducing sequence errors during oligo synthesis.

Several host proteins act at the replication fork to prevent the introduction of replication errors. One of the main molecular repair systems at work is MMR that recognizes and eliminates mismatches (Hsieh, 2001). Introducing defects in this system proved to enhance the efficiency of oligo-mediated recombineering over 100-fold (Costantino and Court, 2003; Wang et al., 2009). Nonetheless, MMR perturbation leads to an accumulation of undesired secondary mutations. To avoid the use of such a background, the use of chemically modified bases (e.g., 2’-fluoro-deoxyuridine, 5-methyl-deoxycytidine, 2,6-diaminopurine or iso-deoxyguanosine), in synthetic oligos proved to lower the occurrence of mismatch detection and repair by MMR, thus improving the efficiency of OMAR up to 20-fold when compared to oligos with standard bases (Wang et al., 2011). Another way to evade MMR is by improving the design of mutating oligos (Sawitzke et al., 2011). Several parameters showed to improve the frequency of oligo-mediated recombination: incorporation of C/C mismatches (a mismatch not recognized by many MMR systems) near a desired base change, or substitution of four or more consecutive bases, since MMR does not recognize multiple mispairs. Nevertheless, introduction of additional changes is not always feasible or desirable as it can alter gene or protein function. Alteration of four or more wobble positions of adjacent codons could help improve oligo recombineering efficiency without altering the protein sequence, though altered mRNA stability and/or protein expression might still come into play (Sawitzke et al., 2011). Recently, the use of dominant-negative mutator alleles of conserved DNA MMR proteins (e.g., MutS and MutL) was implemented in genome engineering. Importantly, controlled expression of a mutator allele aided the transient inhibition of MMR *in trans* only during the recombination process and allows recombineering and efficient OMAR in wild type cells (Nyerges et al., 2016; Bubnov et al., 2018).

Other host recombination functions have generally no effect in OMAR, except when limiting concentrations of oligos are used (∼30 molecules/cell). In this case, significant single-stranded exonuclease-mediated degradation of oligos has been observed, thus affecting the frequency of recombination (Sawitzke et al., 2011). However, recombination frequencies also decreased when modifications were introduced within the last 9 nt. of the 5′ or 3′- oligo end (Sawitzke et al., 2011), providing additional evidence of the occurrence of nuclease degradation at the replication fork, even when making use of an optimal oligo concentration. Another way to prevent oligo degradation by endogenous endonucleases is by protecting the terminal bases with phosphorothioate bonds (Wu et al., 2005). The allelic replacement efficiency increased up to 2-fold when protecting the 5’-oligo end by phosphorothioate bonds (Wang et al., 2009). Of note, although at lower but still detectable frequency of recombination [1 × 10^–4^ recombinants per viable cell (Swingle et al., 2010b)], the use of ssDNA oligos in the absence of any phage encoded functions attained targeted chromosomal modifications in *Escherichia coli*, *Pseudomonas syringae*, *S. enterica*, *Shigella flexneri* (Swingle et al., 2010b), *Shewanella oneidensis* (Corts et al., 2019) and *Legionella pneumophila* (Bryan and Swanson, 2011).

## Recombineering-Introduced Genetic Mutations Resulting in Polar Effects

Genes encoding open reading frames (ORFs) are first transcribed to messenger RNA (mRNA), which is either polycistronic, encoding two or more proteins, or monocistronic, encoding a single protein. In bacteria and archaea, polycistronic mRNA transcripts frequently encode functionally related proteins. A transcriptome study in *S. enterica* found approximately 60% of studied genes to be expressed from polycistronic mRNA transcripts (Ramachandran et al., 2014). In fact, bacterial operons were reported to have an average of 1.4–2.1 genes per operon (Yan et al., 2018). Therefore, the polycistronic nature of bacterial mRNA transcripts makes single gene targeting challenging, mainly because of possible perturbations of downstream or upstream gene expression at the transcriptional and/or the translational level. Upon gene targeting, transcription of the introduced selectable marker (e.g., antibiotic resistance cassette) can affect the expression of downstream genes. Even after removal of such a marker, the presence of the resulting scar, although typically restricted in size, could alter gene expression due to the removal or change of regulatory sequences.

To circumvent this problem when constructing gene knock-outs, homology regions have been designed in such a manner to generate single in-frame deletions. The Keio collection of 3985 gene knock-outs in *E. coli* was created this way to minimize effects of the deletions on the expression of downstream or upstream genes, referred to as polar effects (Baba et al., 2006). This strategy consisted of leaving the sequence encoding the last 7 codons of the ORF targeted for deletion intact, thereby leaving the stop codon and possible RBSs of downstream genes in their normal chromosome context. For instance, deletion of the *hplA* gene, encoding a periplasmatic chaperone for outer membrane proteins, was successfully achieved by this in-frame scar approach. This hinted that the previously presumed essentiality of the *hplA* gene was incorrectly inferred because of a polar effect on expression of the downstream essential gene *lpxD*. During the construction of a similar library in *S. enterica* (Porwollik et al., 2014), in-frame deletions were generated leaving the last 5 codons at the 5′- and 3′-ends of the ORFs targeted for deletions intact. Moreover, for the purpose of monitoring polar effects due to the presence of the promoter of the antibiotic resistance markers, kanamycin and chloramphenicol resistance cassettes were used to target the same gene in both sense and anti-sense translation direction, respectively, Porwollik et al. (2014). Normally, when referring to polar effects, downstream gene effects are discussed since these occur far more frequently due to the direction of transcription. To more precisely avoid polar effects introduced by the presence of selection marker genes or residual scar sequences, the implementation of alternative strategies is clearly needed. Alternative approaches to avoid such polar effects are presented in the next section.

## Seamless Recombineering and Scarless Removal of Selection Markers

**FIGURE 4:**
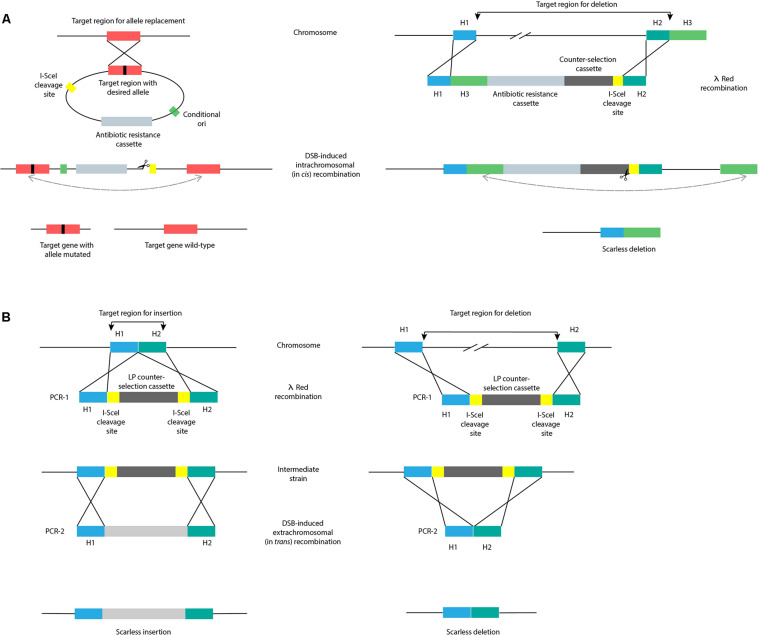
Scarless genome editing by intra- (*in cis*) or extrachromosomal (*in trans*) recombination. **(A)** DSB-induced *in cis* recombination by I-SceI endonuclease, with a mutated version of the target region inserted into the chromosome by a conditional replicating suicide plasmid (left) or with an identical sequence inserted into the chromosome by λ *red* recombination with a linear dsDNA fragment (right). **(B)** DSB-induced *in trans* recombination by I-SceI endonuclease, comprising the initial generation of an intermediate strain by introduction of landing pad (LP) counter-selection cassette (PCR-1) by λ *red* recombination. Subsequent introduction of editing substrates (PCR-2) into the intermediate strain and expression of I-SceI endonuclease stimulates excision of the LP and repair of the DSB by recombination with editing substrates, overall resulting in transformants with an insertion (left) and a deletion (right). H1-H3, homology regions 1-3; DSB, double strand break; LP, landing pad.

Scarless genome editing aims to obtain recombinants devoid of scar sequences. Many of these approaches are based on the use of homing endonucleases, such as I-SceI from *Saccharomyces cerevisiae*. This endonuclease recognizes and cuts an unusually long 18 bp sequence, ensuring a recognition site-specific cleavage. The introduction of an I-SceI recognition sequence in the target region, followed by expression of I-SceI endonuclease from a helper plasmid generates a double strand break (DSB) in the chromosome. This way, either the bacteria cannot survive, or the DSB is fixed by recombination with a sister chromosome, or a provided template. As described below, DSB-induced recombination can involve intra- (*in cis*) or extrachromosomal (*in trans*) recombination (Figure 4). Noteworthy, in case of active transcription and transcription continuation into the inserted mutation cassette, cleavage at the I-SceI site might not be efficient. To overcome this, two successive transcription terminator sequences are often introduced just before the I-SceI specific recognition site (Kim et al., 2014).

DSB-induced *in cis* recombination was first introduced by Posfai et al. (1999) who used a suicide plasmid strategy encoding a mutant copy of the target gene to be introduced in the chromosome, containing both an antibiotic resistance cassette and the I-SceI recognition site (Figure 4A). DSB-induced recombination between direct repeats (wild-type and mutant target gene) was initiated by expressing I-SceI endonuclease from a helper plasmid. Transformants obtained by *in cis* recombination can contain either a copy of the wild-type or mutant gene. In the absence of a DSB, the spontaneous frequency of recombination was calculated to be ∼2 × 10^–3^/generation; with the introduction of a DSB, recombination efficiency increased by 2–3 orders of magnitude (Posfai et al., 1999; Figure 1).

After using a suicide vector, screening for transformants containing the desired mutation is needed. An alternative to the use of suicide plasmids to deliver *in cis* homologous regions is the use of linear DNA in strains expressing λ *red* recombination genes. In this way, deletions up to 117 kbp were generated (Figure 4B; Yu et al., 2008). The linear DNA fragment contains 3 homology regions to the chromosome: H1 and H3 have homology to the upstream and downstream regions targeted for deletion, and H2 has homology to the 3′-end of the region targeted for deletion. The linear fragment is introduced in the chromosome by λ *red*-mediated dsDNA recombination with ∼50 bp homology regions H1 and H2. This step is followed by the introduction of a DSB site within the insertion, allowing for recombination between the duplicated H3 sequences (∼500 bp each). An improvement of this approach uses H3 homology regions with a length of ∼75 bp to perform deletions, shortening the linear fragment length required (Figure 4B; Tas et al., 2015). Moreover, to promote cleavage and elimination of the helper plasmid encoding the λ *red* recombination genes, an I-SceI recognition site can be incorporated in this plasmid, concomitantly allowing assessment of I-SceI endonuclease cutting efficiency (Lee et al., 2009).

DSB-induced recombination with *in trans* substrates (Figure 4B) requires the construction of an intermediate strain. This is achieved by λ *red* recombination of a first PCR fragment (PCR-1) introducing the I-SceI recognition site and an antibiotic resistance cassette into the target region of the chromosome (Figure 4B; Cox et al., 2007). Once the intermediate strain is generated, a second PCR product (PCR-2) is introduced together with a helper plasmid for expression of the I-SceI endonuclease. PCR-2 contains the same homologous flanking sites as PCR-1 and the desired sequence to be inserted, or solely the homologous flanking sites, when a deletion is desired. Only cells where the DSB is repaired by recombination with PCR-2 can survive. The landing pad (LP) strategy (Tas et al., 2015) uses *galK* and *tetA* genes flanked by I-SceI recognition sites to select for the intermediate strain generated and to counter-select DSB-induced recombination and consequent loss of the marker. Large fragment insertions (6.5 kbp) were achieved in the second recombination step with efficiencies ∼75–97% depending on the target site. Moreover, OMAR-assisted LP replacement was used to delete 6 kbp with an efficiency of 60% for lagging oligos, but only 10% for leading oligos (Tas et al., 2015). A variant of the LP technique delivers the LP construct in a plasmid instead of a linear construct to avoid the use of electroporation (Wei et al., 2017). In this study, an alternative endonuclease from *Chlamydomonas reinhardtii*, I-CreI, was used to catalyze the *in vivo* excision of the LP from the plasmid. Similar to the extended 18 bp I-SceI recognition sites, I-CreI recognition sites are 22 bp in length.

Another scarless approach termed gene gorging (Herring et al., 2003) claims to avoid the use of selection markers as the replacement occurs in 1–15% of transformed cells (Figure 1), and this way, the direct screening for mutants containing deletions is in principle permitted. Here, instead of transforming cells with linear DNA, the recombineering template is delivered as a plasmid and co-transformed with another helper plasmid encoding arabinose-inducible λ *red* recombination genes and I-SceI endonuclease. Upon selection of co-transformants, colonies are grown in media containing arabinose and the linear fragment is generated and integrated in the chromosome by I-SceI endonuclease restriction and λ *red* recombineering, respectively. A similar scarless approach (Gerlach et al., 2009) to the one described in Figure 4B, used the insertion of the *tetA* and *tetR* genes providing tetracycline resistance, followed by λ *red* recombination with ∼80 bp complementary oligos in the absence of a I-SceI cleavage step. The oligos were annealed to form dsDNA before electroporation and then, depending on how the flanking homology sites were chosen, an in-frame deletion or site-directed mutagenesis was achieved. When using I-SceI DSB-induced recombination, 96% of the tested clones contained the correct modification (Cox et al., 2007), while leaving I-SceI out resulted in 80–90% correct clones in case of site-directed mutagenesis, but only 33% of correct deletions (Figure 1).

## CRISPR-Cas Gene Targeting Assist Bacterial Recombineering

The successful implementation of selection-free recombineering strategies depends on their editing efficiencies, which can be achieved by increasing the frequency of chromosomal modification to an extent where edited cells represent a significant number of colonies in the outgrowth plate. Another way of increasing the percentage of edited cells in the outgrowth plate is by simply eliminating non-edited cells. In this way, the recently identified prokaryote adaptive immune CRISPR-Cas9 (clustered regularly interspaced short palindromic repeats and its associated protein, Cas9) system can assists bacterial recombineering as the specificity of sequence-specific nucleases can be programmed by small RNA molecules (Barrangou et al., 2007; Jinek et al., 2012). Following identification of these enzymes, CRISPR-Cas nucleases have been broadly used as a genome editing tool in a variety of organisms including bacteria (Feng et al., 2013; Jiang et al., 2013; Wang et al., 2013; Nayak and Metcalf, 2017). CRISPR-Cas nucleases are dsDNA endonucleases that are directed by single guide RNA (sgRNA) to perform sequence-specific cleavages. sgRNA target sequences are termed protospacers, and their cleavage by CRISPR/Cas nucleases essentially requires a triplet sequence downstream of the protospacer termed protospacers-adjacent motifs (PAM), e.g., NGG for Cas9 nuclease from *Streptococcus pyogens*. This requirement does not represent a major limitation for gene editing with *S. pyrogens* Cas9 in *E. coli*, as greater than 400,000 GG doublets are present in the *E. coli* genome (Keseler et al., 2013). To target Cas9 to protospacers, sgRNA is typically encoded in a CRISPR array consisting of short repeated sequences separated by unique spacers that upon processing generates CRISPR RNA (crRNA) to be loaded in Cas9 in a process dependent of a trans-activating crRNA (tracrRNA) and RNAseIII. Specific design of crRNA sequences enables gene-specific Cas9 endonuclease targeting and dsDNA cleavage (Barrangou, 2012), overall making CRISPR very useful for recombineering by specifically targeting non-recombinants (Barrangou et al., 2007; Jinek et al., 2012).

**FIGURE 5:**
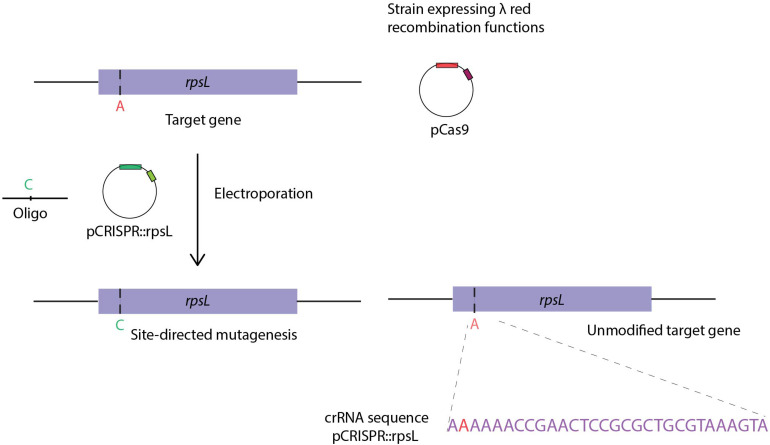
CRISPR-Cas9 based bacterial recombineering. Site-directed mutagenesis of the *rplL* gene by use of an editing oligo in the HME63 strain and an inducible plasmid encoding PCas9 and λ *red* genes. Co-transformation of the editing oligo with the pCRISPR:rpsL plasmid, the latter harboring the crRNA sequence directs Cas9 endonuclease targeting of non-edited cells. Figure adapted from “RNA-guided editing of bacterial genomes using CRISPR-Cas systems” (Jiang et al., 2013).

Jiang and coworkers adapted Cas9 selection for genome editing in bacteria (Jiang et al., 2013). More specifically, Cas9 was constitutively expressed in HME63, a recombineering efficient *E. coli* strain expressing the λ *red bet* gene from the chromosome (Costantino and Court, 2003). A two-plasmid system, pCas9 and pCRISPR, encoding Cas9 and carrying the array of CRISPR spacers, respectively, was used to achieve ssDNA oligo assisted point mutagenesis of *rpsL* with an editing oligo. Co-electroporation of the editing oligo with the pCRISPR plasmid containing CRISPR array with crRNA sequences guided Cas9 cleavage of the wild-type but not the mutant *rpsL* gene (Figure 5). The efficiency of OMAR was increased from ∼0.005 to ∼65% when combined with Cas9 targeting of non-modified chromosomes to eliminate non-edited bacteria (Jiang et al., 2013). Since then, other studies reported on the combined use of Cas9 selection and recombineering in *E. coli*. A three-plasmid system approach in the *E. coli* DH5α strain, reported the use of pCas9 and pCRISPR plasmids (Jiang et al., 2013), as well as pKD46 to deliver λ *red* recombination genes *in trans* (Pyne et al., 2015). Besides single point mutagenesis, other modifications such as large deletions (up to ∼19.4 Kbp) were achieved by combining recombineering using dsDNA editing substrates with Cas9 selection, generally achieving efficiencies ranging from few percentages to about 50% of the colonies following outgrowth. Once successful genome engineering is achieved, the curing of the original helper plasmids is desired, particularly when multiple chromosomal manipulations are performed in the same strain. For example, a plasmid carrying sgRNA sequences, the λ *red* genes and a conditional replicating origin can be cured at non-permissive temperatures of 37°C (Reisch and Prather, 2017). Moreover, plasmid pCas9cr4 elimination was effectively achieved by p15A ori Cas9-directed cleavage, by delivering pKDsg-p15A (Reisch and Prather, 2017). Another nuclease, CRISPR-Cas12a, was also successfully coupled to recombineering in *E. coli*, as comparable efficiencies to those reported for Cas9 were observed when attempting to perform site-directed mutagenesis of *lacZ* or gene specific deletions (∼1000 bp). Although to obtain high efficiencies, and despite co-expression of the Gam, Exo and Bet proteins, efficient insertion with this system required recombineering substrates with 500 bp flanking homologies (Yan et al., 2017).

Taken together, CRISPR-Cas specific gene targeting enhances the efficiency of recombineering, making it a preferred strategy for introducing multiple genome edits as it does not require recycling of selection markers (Jiang et al., 2015; Ronda et al., 2016). The main inconvenience of these strategies is incompleteness of Cas9 targeting of wild-type non-edited cells, referred to as escapers, resulting in an increased background of non-edited cells on the outgrowth plate. A recent study based on the oligo-assisted point mutagenesis of *rpsL* coupled to CRISPR-Cas9 (Cui and Bikard, 2016) showed that *E. coli* can survive Cas9 cleavage at certain targets by triggering the SOS response, which results in repair of the dsDNA break with a sister chromosome by homologous recombination. To improve efficiency of Cas9 targeting of non-edited cells, the Gam protein from phage Mu known to block DNA repair was expressed (Shee et al., 2013). Escapers can also result from spontaneous mutations in crRNA, PAM or protospacer sequences, preventing sequence-specific CRISPR-Cas9 nuclease targeting (Jiang et al., 2013).

Since recombineering coupled to CRISPR-Cas strategies was only implemented in recent years, protocols have typically been developed in strain-specific manners and optimized for a single or few target(s) (Jiang et al., 2015; Ronda et al., 2016). A robust method widely applicable to different chromosomal targets is limited by the broad variation in the on-target activity of sgRNAs observed (Guo et al., 2018). To overcome this, a comprehensive library analyzing 70,000 sgRNAs targeting different regions in the *E. coli* chromosome distinguished highly active sgRNA sequences from “protected” genome sites with weak sequence-specific CRISPR-Cas9 specificity. From this extensive map, a tool to design optimal sgRNA sequences was developed to facilitate further assistance of CRISPR-Cas9 in recombineering (Guo et al., 2018). Nevertheless, further efforts to elucidate chromosomal features and mechanisms “protecting” specific targets are needed to optimize CRISPR-Cas9 specificity and to obtain similar efficiencies over the whole chromosome. Finally, other drawbacks in the implementation of CRISPR-Cas9 assisted recombineering such as Cas9 cytotoxicity are discussed in Vento et al. (2019).

## λ *Red*-Based Recombineering in Other Bacterial Species

While inherently, non-transformable bacteria cannot be subjected to recombineering, adapting this approach to transformable non-*E.coli* hosts is still challenging as λ *red* and Rac recombineering functions do not display the same efficiency across bacteria. Besides manipulation of the RecA-RecBCD dependent recombination pathways as a useful alternative in case of λ *red* incompatibility, better success for recombineering of non-*E. coli* bacteria has been to employ bacteria-specific phage homologous recombination systems (van Kessel and Hatfull, 2007, 2008; Swingle et al., 2010a; Yin et al., 2015, 2017, 2019; Lee et al., 2017; Murphy et al., 2018; Corts et al., 2019). For example, inducible expression of the distantly related RecE and RecT mycobacteriophage Che9c *gp60* and *gp61* genes catalyzed the deletion of different genes in *M. smegmatis* and *M. tuberculosis*, using dsDNA substrates with ∼500 bp flanking homologies (van Kessel and Hatfull, 2007). Later, Che9c *gp61* was shown to promote ssDNA recombineering using homologies as short as 50 nt oligos (van Kessel and Hatfull, 2008). In a recent study, *gp61*-based oligo recombineering was used to introduce an *attP* site into the chromosome target site. Simultaneous co-expression of the phage Bxb1 integrase induced site-specific recombination between *attP* and *attB* sites, the latter being delivered on a non-replicating plasmid with an antibiotic selection marker attaining insertion and gene editing (Murphy et al., 2018). This ingenious workaround approach termed oligonucleotide-mediated recombineering followed by Bxb1 integrase targeting (ORBIT) overcomes the use of lengthy flanking homologies needed when using dsDNA editing substrates. Following deletion formation, curing of the drug marker by plasmid excision is achieved by expressing the phage Bxb1 site-specific excision system, which includes both the phage integrase and directionality factor gp47. Nevertheless, ORBIT leaves the antibiotic resistance markers or an *attP* site (48 bases) in case of insertions and deletions, respectively, Murphy et al. (2018).

Another example of the utility of an endogenous phage recombination system in a non-*E. coli* bacteria is the use of W3 Beta recombinase from a *Shewanella oneidensis* prophage, a homolog of λ *red* Beta sharing 55% identity. This protein catalyzes recombination with ssDNA oligos containing 40 nt homology arms, and was also functional in *E. coli* with similar efficiencies as compared to λ *red* Beta (Corts et al., 2019). The use of recombinase proteins encoded in the SXT mobile genetic element was also reported in the marine bacterium *Vibrio natriegens*. Here SXT-Beta and SXT-Exo share 43.6 and 21.5% identity with their λ- homologs, respectively. Even though SXT-Beta catalyzed recombination reactions with 90 nt ssDNA oligos, recombination with dsDNA editing templates (flanked by long-homology arms of 500 bp) requires the additional expression of SXT-Exo and λ-Gam, as no homolog of the later protein was identified (Lee et al., 2017).

The Plu2934, Plu2935, and Plu2936 phage proteins encoded by the *red*-like operon Pluγβα (functional analogs of Redγ, Redβ en Redα) was shown to be effective in *Photorhabdus luminescens*. While Plu2934 (or Plu-Gam) was functional in *E. coli*, λ-Gam was not active in *P. luminescens* and Plu2934 recombination efficiencies obtained were higher in its endogenous host *P. luminescens* than in *E. coli* (Yin et al., 2015, 2017). The implementation of Plu-Gam protein was further tested in *Pseudomonas* strains aiming to establish a broadly applicable recombineering protocol in this large genus (Yin et al., 2019). More specifically, Plu-Gam, and λ-Gam improved the recombineering efficiency of RecTE_*Psy*_, a RecET-like operon from *Pseudomonas syringae* (Swingle et al., 2010a), as well as the λ-*red*-like BAS operon from *Pseudomonas aeruginosa* phage Ab31 (Yin et al., 2019) in *P. aeruginosa* and *P. fluorescens*, but not in *P. putida* or *P. syringae*. Of note, while λ *red* and Plu operons were inefficient to promote recombineering in *Pseudomonas*, the BAS operon was functional in all of the *Pseudomonas* strains mentioned above. Interestingly, in addition to the *orf38* (or B) and *orf37* (or A) homologs of λ *red bet* and *exo*, respectively, the AS operon contains *orf36* (or S), a ssDNA binding (SSB) protein that was shown to improve the recombination efficiency of BA and RecET in *E. coli*, but not λ *red* functions (Yin et al., 2019).

These studies clearly indicate the dependence of phage recombineering functions on host-specific machinery (Datta et al., 2008), in line with reported observations on similar efficiencies of λ *red* recombineering in related bacteria such as *S. enterica* (Stanley et al., 2000; Czarniak and Hensel, 2015), *Shigella flexneri* (Shi et al., 2003; Wang Y. et al., 2014), *Klebsiella pneumoniae* (Wei et al., 2012; Guo et al., 2013), *Yersinia enterocolitica* (Trulzsch et al., 2004), *Yersinia pestis* (Sun et al., 2008), and *Zymomonas mobilis* (Khandelwal et al., 2018). In these studies, λ *red* recombineering genes were expressed from pKD46 (Datsenko and Wanner, 2000) or pSIM (Datta et al., 2006) plasmids, from P_*BAD*_ or P_*L*_ promoters, respectively. In particular, attempts to use pKD46 in *Escherichia albertii* were unsuccessful, while pSIM λ-*red* expression was effective for achieving chromosomal mutations (Egan et al., 2016), an observation presumably due the higher levels of expression of the λ *red* genes when expressed from pSIM, when compared to pKD46 (Datta et al., 2006). Noteworthy, in these bacteria, the lengths of flanking homologies were similar to those reported for *E. coli*. For completeness we also refer to Murphy (2016), where more examples on transferring the recombinogenic potential of λ *red* genes to other bacteria are discussed.

In addition to phage origin, expression of phage recombineering functions often require host compatible plasmid systems. A representative example of this is the use of λ *red* recombineering expression system in *Agrobacterium tumefaciens*. More specifically, the arabinose inducible promoters (i.e., pBAD) commonly used for λ *red* genes expression, could not be implemented in *Agrobacterium tumefaciens* as this promoter did not work well in this bacterial context; as such, a tetracycline inducible promoter was used instead (Hu et al., 2014). While an initial study in *Pseudomonas aeruginosa* reported on the requirement of long homology flanking regions of 400-600 bp when expressing λ *red* recombineering genes (Lesic and Rahme, 2008), expression of λ *red* recombineering genes from another plasmid, pRKaraRed (Liang and Liu, 2010) (with *oriV* and *trfA* regions to support plasmid replication and stability in *P. aeruginosa*) showed homology flanking region requirements of only 50 bp. In some cases, the expression of λ *red* recombineering genes was proven to be toxic, as shown for the enterobacteriaceae *Pantoea ananatis* (Katashkina et al., 2009), requiring the use of mutant strains to express λ *red* genes (Katashkina et al., 2009). Adaptation of Rac recombineering was proven successful in case of *Zymomonas mobilis* (Wu et al., 2017), *Acinetobacter baumannii* (Tucker et al., 2019), *Corynebacterium glutamicum* (Huang et al., 2017) and *Pseudomonas putida* (Choi and Lee, 2020) among others.

## Conclusion

Reverse genetic approaches to decipher gene function have significantly improved over the years with the development of highly efficient recombineering approaches. In this way, the realization of successful transformants was significantly improved as it merely required a one-step electroporation protocol of simple PCR products or synthetic oligos. Furthermore, prior subcloning of the mutant allele containing the desired genetic modification in appropriate vectors (i.e., suicide vectors) was circumvented. Selection of a suitable recombineering strategy depends on the intended modification. For instance, when aiming to insert DNA sequences, the size of the insert must be considered to choose the proper editing substrate. In particular, short insertions can be achieved by ssDNA oligos [up to 60 bp – see Murphy et al. (2018)], while the introduction of longer sequences, in cases of gene tagging or reporter fusions, required dsDNA recombineering substrates. Besides this limitation, OMAR approaches obtain a higher efficiency than dsDNA recombination and are scarless, as they avoid the use of selection markers; oligo recombineering is also mechanistically better understood. On the other hand, the development of scarless approaches based on eliminating non-edited background by specific endonucleases (e.g., I-SceI), or more recently, the use of CRISPR directed Cas9 endonuclease, increased the percentage of positive transformants to the point where, as in OMAR approaches, the use of selection markers is often not mandatory (Figure 1), making these approaches preferable over those relying on (counter) selection of introduced markers. In contrast to the use of other specific endonucleases (e.g., I-SceI) for the elimination of non-edited background, the use of CRISPR-Cas endonucleases do not require the *a priori* introduction of specific recognition sequences for cleavage (e.g., the I-SceI recognition sequence). The implementation of CRISPR-Cas endonucleases however remains critical due to the outgrowth of wild-type survivors, also termed escapers. Furthermore, reported frequencies of escapers vary greatly dependent on the target gene, hinting that future research efforts need to focus on elucidating the molecular basis of these findings as to increase the robustness and wider applicability of CRISPR-Cas selection in assisting recombineering. Finally, while recombineering strategies described in this review mainly provide an overview of currently existing strategies to attain different genetic engineering in *E. coli* and *S. enterica* for reverse genetics studies, λ *red* recombineering and RecET phage proteins are not always as efficient or even operational as their usage failed in many distantly related *Escherichia coli* strains. As such, future recombineering efforts may help to overcome remaining pitfalls and drawbacks and extend the use of recombineering to other bacteria.

## Author Contributions

UF and PV conceived the theme and content of the review. UF, KG, and PV wrote the manuscript. All the authors have revised and approved the manuscript.

## Conflict of Interest

The authors declare that the research was conducted in the absence of any commercial or financial relationships that could be construed as a potential conflict of interest.
